# Tracking SARS-COV-2 Variants Using Nanopore Sequencing in Ukraine in Summer 2021

**DOI:** 10.21203/rs.3.rs-1044446/v1

**Published:** 2021-11-30

**Authors:** Anna Yakovleva, Ganna Kovalenko, Matthew Redlinger, Mariia G. Liulchuk, Eric Bortz, Viktoria I. Zadorozhna, Alla M. Scherbinska, Joel O. Wertheim, Ian Goodfellow, Luke Meredith, Tetyana I. Vasylyeva

**Affiliations:** University of Oxford; University of Cambridge; University of Alaska Anchorage; State Institution “L.V. Gromashevsky Institute of Epidemiology and Infectious Diseases of National Academy of Medical Sciences of Ukraine”; University of Alaska Anchorage; State Institution “L.V. Gromashevsky Institute of Epidemiology and Infectious Diseases of National Academy of Medical Sciences of Ukraine”; State Institution “L.V. Gromashevsky Institute of Epidemiology and Infectious Diseases of National Academy of Medical Sciences of Ukraine”; University of California San Diego; University of Cambridge; University of Cambridge; University of California San Diego

**Keywords:** SARS-COV-2, Nanopore, Ukraine, COVID-19

## Abstract

Since spring 2020, Ukraine has experienced at least two COVID-19 waves and has just entered a third wave in autumn 2021. The use of real-time genomic epidemiology has enabled the tracking of SARS-CoV-2 circulation patterns worldwide, thus informing evidence-based public health decision making, including implementation of travel restrictions and vaccine rollout strategies. However, insufficient capacity for local genetic sequencing in Ukraine and other Lower and Middle-Income countries limit opportunities for similar analyses. Herein, we report local sequencing of 24 SARS-CoV-2 genomes from patient samples collected in Kyiv in July 2021 using Oxford Nanopore MinION technology. Together with other published Ukrainian SARS-COV-2 genomes sequenced mostly abroad, our data suggest that the second wave of the epidemic in Ukraine (February-April 2021) was dominated by the Alpha variant of concern (VOC), while the beginning of the third wave has been dominated by the Delta VOC. Furthermore, our phylogeographic analysis revealed that the Delta variant was introduced into Ukraine in summer 2021 from multiple locations worldwide, with most introductions coming from Central and Eastern European countries. This study highlights the need to urgently integrate affordable and easily-scaled pathogen sequencing technologies in locations with less developed genomic infrastructure, in order to support local public health decision making.

## Introduction

The novel severe acute respiratory syndrome coronavirus 2 (SARS-CoV-2) was first identified in a cluster of pneumonia cases with an unknown cause in Wuhan, China in December 2019 [1, 2]. The virus quickly spread and reached pandemic status by March 2020; with over 243 million cases reported and resulting in over 4.95 million deaths globally at the time of writing [3]. In just under a year since the beginning of the pandemic, several SARS-CoV-2 vaccines have been developed and deployed [4]. Current vaccination efforts have reached an estimated 44.9% of the global population, who have received at least one vaccine dose, however, only 2.3% of vaccine recipients so far reside in low-income countries [4, 5].

Genomic epidemiology has become a state-of-the-art approach for Coronavirus Disease 2019 (COVID-19) outbreak investigations, providing an evidence-based foundation for public health contingency measures such as travel restrictions and national lockdowns [6, 7]. Rapid SARS-CoV-2 genomic sequencing has been instrumental in informing public health systems of novel virus importations, informed the detection of transmission chains for contact tracing, and enabled rapid identification of novel variants of concern (VOC) and variants of interest (VOI) that display altered transmission, immune evasion, and/or epidemiological properties [8–10].

Ukraine, with a population of 42 million people, has reported over 2.4 million cases of COVID-19 and more than 56,000 deaths from March 2020 through September 2021 [3] [11]; whilst an estimated 13% of the total population have received two vaccine doses as of the end of September 2021 [12]. The national COVID-19 response in Ukraine is complicated by the transition from a fully government controlled to an insurance-based healthcare system [13]. It is expected that health and economic consequences due to the COVID-19 pandemic are likely to be severe in Ukraine, which is currently classified as a lower middle-income country [14].

Most recently, Ukraine was one of the few European countries to open its borders during the summer period in 2021, requiring a negative SARS-CoV-2 test pre-departure and purchase of private medical insurance as basis for entry for foreign citizens (https://visitukraine.today/). With the aim of encouraging international tourism and supporting rapid economic recovery, businesses such as restaurants, bars, and nightclubs were permitted to operate at full capacity [15]. Over 300,000 international tourists travelled to the capital city of Kyiv in 2021, a significant increase in number of visits compared to pre-pandemic years, with a total of 86,840 international tourists travelling to Ukraine in 2019 [16–19].

Ukraine has a recent history of outbreaks of infections such as polio and measles, [20, 21], drug-resistant tuberculosis [22, 23], and numerous veterinary pathogens [24], which are sustainably controlled in other countries. Furthermore, despite a high HIV and hepatitis C prevalence, infectious disease genomic epidemiology is currently not integrated into public health decision making in Ukraine [25, 26]. Unlike other European countries, this is due to the unavailability of large-scale genetic sequencing capacity in Ukraine. As a result, during the SARS-CoV-2 epidemic and at the time of writing, sequencing activities were not supported or funded by the government, and SARS-COV-2 genomic epidemiology has not contributed to the public health response in Ukraine.

Currently, only one study on the phylodynamic analysis of the SARS-CoV-2 sequences obtained from Ukrainian patients has been reported, describing the first four months of the pandemic. It showed that the initial rise in the number of COVID-19 cases was likely led by multiple independent introductions of the virus into Ukraine prior to border closures [27], similar to the findings of the importation and circulation of SARS-CoV-2 lineages in the UK [7]. For the first time, our study aimed to build local and sustainable SARS-COV-2 sequencing capacity and genomic epidemiology expertise in Kyiv, Ukraine using the Nanopore MinION sequencing platform. We generated and analysed the most recent SARS-CoV-2 genetic sequences obtained in Ukraine in summer 2021 and integrated our findings with global surveillance data, thus providing an evidence-based framework for future public health decisions in Ukraine.

## Results

### Epidemiology of COVID-19 in Ukraine between March 2020 and September 2021

**Figure 1:**
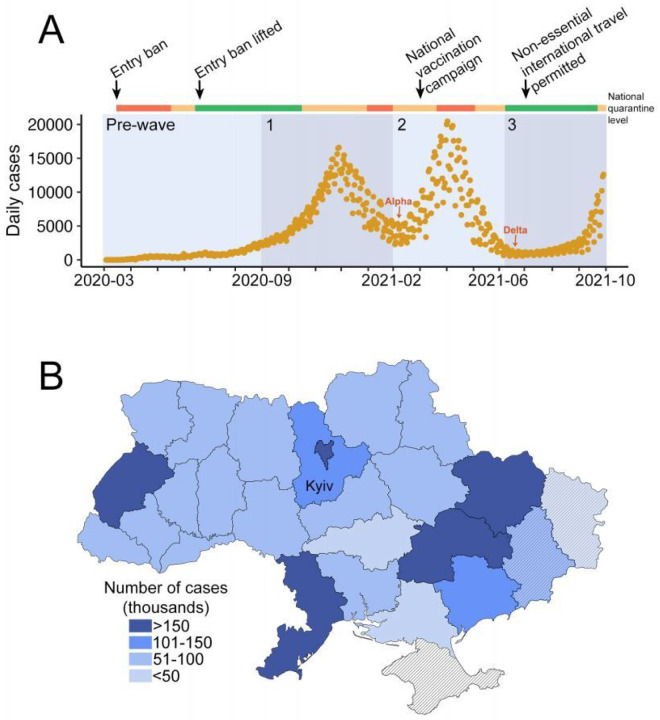
(A) Confirmed COVID-19 cases (orange) reported in Ukraine between March 2020 and September 2021 at the national COVID-19 tracking portal (Four broad timeframes were assigned to indicate the “Pre-Wave” (March to 31 Aug 2020), “Wave 1” (01 Sep 2020 – 31 Jan 2021), “Wave 2” (01 Feb 2021 – 09 Jun 2021) and “Wave 3” (10 Jun 2021 to 30 September 2021). National quarantine measures, and implementation and easing of travel restrictions, are indicated along the colored bar on the top. The colors indicate national quarantine level at the time, with red representing complete national quarantine, orange representing adaptive quarantine with varied levels of restrictions in different regions, and green representing no or few restrictions. Detection of the first Alpha and Delta variants in sequenced samples obtained from GISAID are indicated with red arrows. The graph was plotted in R Studio using the package ggplot2. (B) The distribution of COVID-19 cases by administrative regions in Ukraine. Regions not fully controlled by the Ukrainian government are marked with a striped pattern.

According to the national COVID-19 tracking portal, Ukraine has experienced two waves of the epidemic, one with a peak of 16,494 daily cases on November 27th, 2020 and another daily peak of 20,454 cases on April 3rd, 2021 [5], with an ongoing third wave beginning August, 2021 to the time of writing (Figure 1A). The distribution of cases by region was uneven, with the capital city of Kyiv, and major urban centers in Odesa, Kharkiv, Dnipro, and Lviv regions being most affected, with over 150,000 cases reported in each (Figure 1B). Data from Donetsk and Lugansk regions are likely to be underestimated due to incomplete data availability across these regions, whilst no data was available from Crimea, as reported by Ukrainian national public health organisations.

### SARS-CoV-2 genomic epidemiology in Ukraine

#### SARS-CoV-2 sequencing with MinION

**Figure 2:**
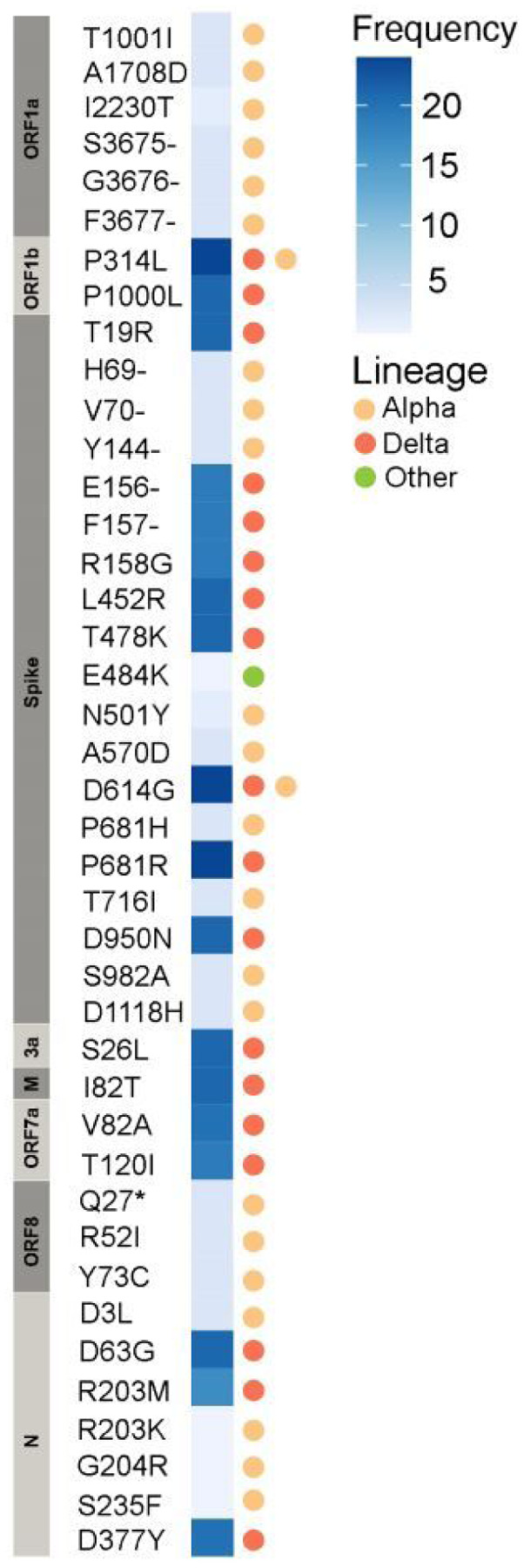
Heat map of frequency of amino acid mutations found in 24 SARS-CoV-2 genomes sequenced from patients in Kyiv, Ukraine, in July 2021. Mutations associated with variant of concern lineages Alpha and Delta are indicated.

Using portable MinION device from Oxford Nanopore Technologies (ONT) and SARS-CoV-2 ARTIC Network workflow, we locally sequenced and analysed a set of clinical samples (nasopharyngeal swabs) (N=24) obtained from 24 individuals (61% female) who tested positive for SARS-CoV-2 by qRT-PCR (cycle threshold (Ct) values ranged 5–23) in July 2021 in Kyiv, Ukraine (Table 1, Supplementary Table S1). We identified two variants of concern (VOC) in our samples: the Delta VOC (B.1.617.2-like) (N=21) and the Alpha VOC (B.1.1.7-like) (N=3) variants. Twenty one out of 24 resulting sequences yielded high quality genomic data, achieving ≥95% genome coverage with read depth of 20X and higher; the other three sequences had 94%, 94% and 91% coverage (samples #10, #22, and #19, respectively). Virus genome analysis identified all common amino acid (aa) mutations which are associated with the Alpha and Delta VOC (Table 1 and Figure 2). Additionally, sample #5, assigned as an Alpha variant, had the E484K mutation (G23012A) in the receptor binding domain (RBD) of the spike (S) protein and the N501S mutation instead of the N501Y mutation commonly found in this position for Alpha variant (Figure 2). More details on the sequence quality and identified mutations can be found in the Supplementary Text.

#### SARS-CoV-2 lineages circulating in Ukraine

**Figure 3:**
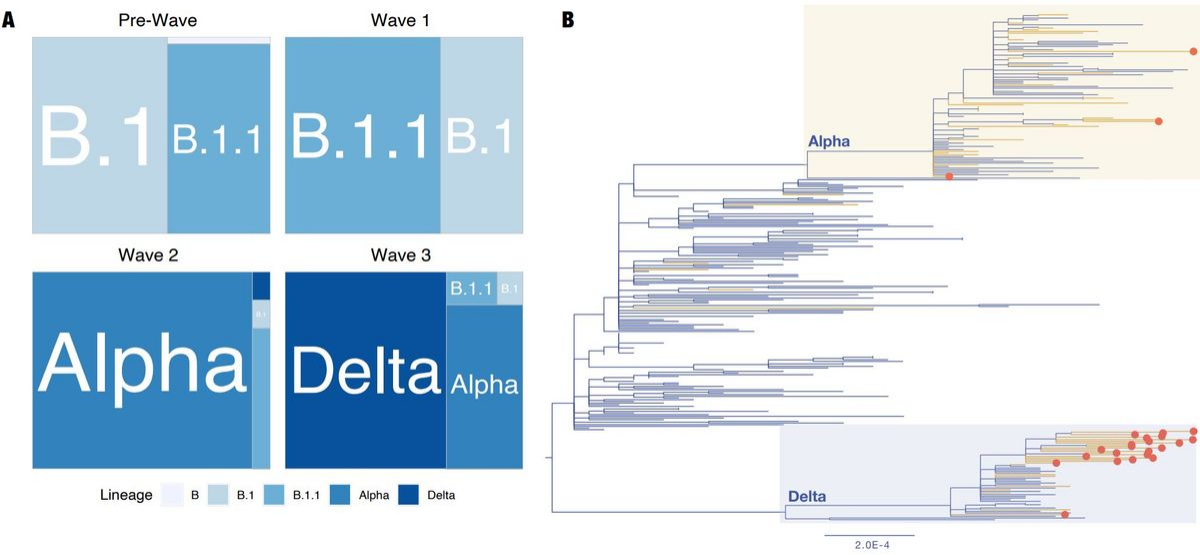
Phylogenetic analysis of SARS-CoV-2 lineages in Ukraine. (A) Relative distribution of dominant SARS-CoV-2 PANGO lineages in epidemic waves of COVID-19 in Ukraine; (B) Maximum likelihood phylogenetic tree representing SARS-CoV-2 genetic diversity in Ukraine. Yellow branches correspond to sequences sampled in Kyiv. Red circles indicate genomes generated in this study by Nanopore sequencing. The bar at the bottom indicates genetic distance in substitutions per site per year. Branch tip labels are not shown for clarity of tree structure.

Next, we analysed SARS-CoV-2 lineage distribution using all available sequences (N=362) obtained from patient samples collected in Ukraine in the GISAID database as of September 30th, 2021 (Supplementary Table S3). Only 355 sequences collected between March 2020 and July 2021 were selected and lineage assignments were carried out using the PANGO nomenclature system (Supplementary Table S2); seven sequences were excluded from the analysis due to poor quality or lack of collection date information. In total, 49 SARS-CoV-2 lineages were designated for 379 sequences from Ukraine: of these, 355 were obtained from GISAID and the majority of which were sequenced abroad (N=251), whilst 24 are the latest available sequences generated locally in this study. The SARS-CoV-2 lineage distribution in Ukraine consisted of four predominant lineages (Supplementary Figure S1): B.1 and its sub-lineages (N=78), B.1.1 and its sub-lineages (N=107), Alpha variant (N=116) (including the B.1.1.7-like sequence with the E484K mutation) and Delta variant (N=77) (Supplementary Figure S1 and Supplementary Table S2). Among the 77 Delta variants, 26 were assigned to nine Delta VOC sub-lineages: AY.4, AY.5, AY.11, AY.16, AY.23, AY.24, AY.26, AY.33 and AY.36. Lineage prevalence varied through the epidemic in the country, consisting of four broad time frames: a “Pre-Wave” (March to 31 Aug 2020) and “Wave 1” (01 Sep 2020 – 31 Jan 2021) dominated by B.1 and B.1.1, “Wave 2” (01 Feb 2021 – 09 Jun 2021) dominated by Alpha VOC and “Wave 3” (10 Jun 2021 to 30 September 2021) dominated by Delta VOC (Figure 3A). Despite the low number of sequences in comparison to confirmed infections (sequence data represent approximately 0.015% of COVID-19 cases), we found a diversity of B.1 and B.1.1, and Delta VOC sub-lineages in Ukraine (Supplementary Figure S1). The closest country of origin for all identified lineages is indicated in the Supplementary Table S2. Only 280 of the Ukrainian sequences on GISAID were complete genomes and of high enough quality to be included in the phylogenetic reconstruction of the SARS-CoV-2 genetic diversity in Ukraine, together with the 24 sequences generated in this study (Figure 3B).

#### Phylogeographic analysis of the Delta variant sequences

We included 1,733 Delta variant sequences in the phylogeographic analysis to understand the timing and origin of Delta variant introductions in Ukraine. This included 56 Ukrainian sequences: 21 Delta sequences generated in this study, 11 sequences from Crimea, and 24 other Delta sequences from Ukraine; and 1,677 sequences from other countries. Our analysis indicated at least 34 separate introductions into the country: most of the Ukrainian sequences were singletons on the global Delta diversity tree, but we also found 6 pairs, two clusters of five sequences, one cluster of three sequences, and one cluster of seven sequences (Supplementary Figure 2). We found the highest number of introductions from Turkey (four individual introductions) and at least one importation event from each of the Baltic countries, Slovenia, Nigeria, Jordan, Russia, Finland, Czech Republic and United Arab Emirates. The TreeTime mugration analysis was not able to resolve the ancestral location for ten separate introduction events.

## Discussion

Ukraine has experienced three COVID-19 epidemic waves between the identification of the first case on March 3rd 2020 and at the time of writing. Compared to other European countries, the epidemic waves in Ukraine were lagging behind by several months as Ukraine did not experience many cases in spring 2020, likely due to a combination of a timely lockdown and a restriction of movement policy [27, 28]. The border closure was applied to both foreign citizens and Ukrainian nationals in March 2020, three weeks after the first case was diagnosed in the country. However, lower numbers of registered cases in Ukraine may also be attributed to a large number of infections likely being undiagnosed due to limited testing and diagnostic capacity in spring 2020.

When most of the world was approaching the second wave of the epidemic in September 2020, the number of COVID-19 cases in Ukraine started to grow quickly, resulting in the first epidemic wave in the country. Just as the epidemic waves in Ukraine followed a similar trajectory as in Western European countries, but with a few months delay, they are also dominated by the same VOCs. As such, the second wave of the epidemic in Ukraine, which started in February 2021, was dominated by the Alpha VOC, first identified in the UK in September 2020 [29]. We now report evidence that the beginning of the third wave of the epidemic is driven by the Delta VOC, which was first identified in India in December 2020, and is currently remaining dominant amongst the European Economic Area countries [30].

Unlike the strict measures in the beginning of the epidemic, no travel restrictions were imposed in summer 2021. This resulted in a tourism boom in Ukraine when almost four-times more tourists visited Ukraine in summer 2021 than in summer 2019 [18]. Increased movement likely contributed to the recently rising number of COVID-19 cases in the country, which has now reached the third wave in autumn 2021. Our analysis showed that the Delta variant, which has dominated in Ukraine since June 2021, has been introduced into Ukraine from multiple geographic locations. While our phylogeographic analysis suggested Delta VOC sub-lineages were introduced from nearby countries, such as Turkey, Russia, and Eastern European nations, we found evidence for introduction of sub-lineages from more distant locations in Africa and the Middle East. Interestingly, no introductions were found from the ten countries with the highest number of Delta cases (see Methods). Such vast geographic spread of virus introductions into the country is likely the result of the absence of travel restriction measures in Ukraine and the increase in the tourist flow. Though we found limited evidence for sustained local transmission of Delta variants in our analysis – most introductions were singletons and were not part of larger Ukrainian clusters – this is likely attributed to a very low number of sequences currently available from Ukraine. Importantly, although we tried to find a balanced approach to sub-sampling of the global Delta variant diversity for our phylogeographic analysis, such inference is complicated by uneven testing and sequencing efforts across the globe, and by the fact that identical sequences can be found in various countries considered in the analysis. Thus, results of our phylogeographic analysis should be interpreted with caution.

Large-scale genomic sequencing of SARS-CoV-2 has enabled global surveillance of pandemic dynamics but is yet to become part of the public health response in Ukraine. To enable development of genomic epidemiology expertise and sequencing capacity in Ukraine, for the first time, we utilised Oxford Nanopore MinION technology in combination with ARTIC Network protocols to locally sequence 24 SARS-CoV-2 genomes collected from patient samples in Kyiv, Ukraine. While most of the genomes were complete and of high-quality, some of the sequences produced in this study had lower than optimal genome coverage (<95%), likely due to poor sample quality or low amplification efficiency where the amplicons are not equally amplified across all regions of the genome. The ARTIC V3 primer set was used, which has been previously reported to be associated with the amplicon 72 (S gene) dropout in the Delta variant sequences as observed here. This prompted the development of the ARTIC V4 primer set compatible with the novel mutations [31]. The second major amplicon dropout reported here (amplicon 64 in ORF1ab) was likely due to a similar issue in primer compatibility.

Genomic epidemiology integration within public health response allows tracking of variants and mutations that potentially affect vaccine efficacy. For example, the E484K mutation in the S protein, an escape mutation that shows evidence of impacting the immune response and likely reducing vaccine effectiveness [32], was identified in one Alpha variant sequence in our analysis. This mutation is rare for this lineage and is usually present in other VOC (Beta, Gamma, Zeta, and Eta) [33]. As of October 3rd 2021, the E484K mutation was identified in 3,319 (0.3%) of all Alpha sequences in GISAID and 0.2% of sequences from Europe. The N501S mutation in S, another mutation found in one of our Alpha variant sequences, was also reported to increase receptor-binding domain (RBD) stability and RBD-ACE2 (angiotensin converting enzyme 2) binding affinity compared to the original Wuhan-Hu-1 isolate but was predicted to have a neutral effect on protein [34]. Furthermore, the same sample sequence had the Y144- deletion in the S protein associated with antibody escape [35]. Tracking of the prevalence of these mutations in Ukrainian SARS-CoV-2 viral population is of immense importance for the country’s vaccine strategy going forward.

Vaccination in Ukraine against COVID-19 is among the lowest in Europe, with 5.6 million people fully vaccinated (13% of the population) and 17% partially vaccinated, as of the end of September 2021 [12], although vaccine uptake is slowly increasing [36]. Without domestic vaccine manufacturing, Ukraine has relied on donations and external sourcing, for example, the WHO COVAX program that supplied 4.2 million doses total of AstraZeneca AZD1222 ChAdOx1, Pfizer-BioNTech BNT162b2 mRNA, and Moderna mRNA-1273 vaccines [37, 38]. Vaccination began in February 2021, with half of the vaccine doses have been adminisered primarily to adults aged 20–49, and the remainder to those over 50+ years, with a small proportion of teenagers. However, the Delta VOC has driven hospitalizations and deaths in unvaccinated patients in other countries such as the United States [39], a pattern that may be repeating in Ukraine.

The Delta variant is characterized by increased transmissibility and virulence, and marginally lower vaccine efficacy (VE) of multiple vaccines against symptomatic COVID-19 [4, 39]. In combination with waning immunity against SARS-CoV-2 [4, 40, 41], the WHO now recommends booster vaccination to at-risk populations to enhance protection against the Delta VOC [37]. Despite the evidence that Delta variants are the dominant lineage in the third COVID-19 wave in Ukraine (Figure 3A), with rapidly increasing cases (Figure 1) and deaths (WHO 2021a), no booster vaccinations are being offered in Ukraine due to the limited availability of VOC surveillance to provide evidence of current virus circulating patterns, as well as access to more vaccine doses in this lower-middle income country. As we report in this study, there is a pressing need for input from genomic epidemiology to inform public health policy of the threat of Delta variants. Availability of national genomic surveillance earlier in the COVID-19 epidemic may have influenced better interventions, such as alternative timing of lifting of local restrictions and possibility of international travel, thus slowing the spread of the Delta variant in Ukraine. Future investment in establishing a sustainable and local genomic surveillance program for SARS-CoV-2 and other prevalent pathogens will ensure evidence-based public health decision-making in Ukraine.

## Methods

### Ethics

All the experiments and analyses were performed in accordance with the relevant guidelines and regulations. All study protocols were approved by The Committee on Medical Ethics and Deontology of the State Enterprise “Institute of Epidemiology and Infectious Diseases named after L.V.Gromashevsky of the National Academy of Medical Sciences of Ukraine”, Kyiv, Ukraine (reference number: No.5 dated 07 October 2021). The requirement for written informed consent was waived by The Committee on Medical Ethics and Deontology of the State Enterprise “Institute of Epidemiology and Infectious Diseases named after L.V.Gromashevsky of the National Academy of Medical Sciences of Ukraine”, Kyiv, Ukraine (reference number: No.5 dated 07 October 2021) since we used anonymized and deidentified samples provided for clinical testing.

### Sample collection

The State Institution “L.V. Gromashevsky Institute of Epidemiology and Infectious Diseases of National Academy of Medical Sciences of Ukraine” in Kyiv, Ukraine, obtained a randomised collection of COVID-19 patient nasopharyngeal swab samples from a private laboratory biobank. Samples were collected in Kyiv between July 20–25, 2021 and infection was confirmed by qRT-PCR using Abbott real-time SARS-CoV-2 amplification kit at the L.V. Gromashevskiy Institute. Following confirmation of SARS-CoV-2 infection, samples were chosen for full length genome sequencing if the Ct value was lower than 30.

### Oxford Nanopore SARS-CoV-2 sequencing

Sequencing was performed at the L.V. Gromashevskiy Institute of Epidemiology and Infectious Diseases in August 2021. Viral RNA was extracted from 140 μL patient sample using the QIAamp Viral RNA Mini kit according to the manufacturer’s instructions (Qiagen) and samples were sequenced using the NEBNext ARTIC SARS-COV-2 Companion Kit for Oxford Nanopore (New England BioLabs) with ARTIC nCoV-2019 v3 primers (https://www.protocols.io/view/ncov-2019-sequencing-protocol-v3-locost-bh42j8ye) as per the manufacturer’s instructions. Briefly, cDNA was generated using random hexamers, and the full-length genome was amplified with a multiplex PCR approach consisting of 400bp overlapping amplicons. Subsequently, samples were purified with SPRI beads and uniquely labelled using the Native Barcoding Expansion kits Oxford Nanopore EXP-NDB104 (1–12) and EXP-NBD114 (1–24). Finally, libraries were prepared, each containing 24 samples, by ligation of Oxford Nanopore sequencing adapters and quantified using Quantus Flurometer (Promega). Libraries were sequenced using the MinION flow cells version 9.4.1 (Oxford Nanopore Technologies).

### SARS-CoV-2 genome assembly

Genomes were assembled using the ARTIC bioinformatics pipeline with a 20X minimum coverage across all genomic regions. First, basecalling was performed with guppy 5.0.11 using the dna_r9.4.1_450bps_sup.cfg model followed by demultiplexing with guppy 5.0.11 for barcode kits EXP-NBD104 and EXP-NBD114. Then read filtering was performed with Artic Guppyplex allowing for read length between 400–700 nucleotides. Finally, ARTIC Minion nanopolish pipeline was run with the option to normalise to 200. All sequences obtained are available on GISAID EpiCov database under accession numbers: EPI_ISL_5852904, EPI_ISL_5852905, EPI_ISL_5852906, EPI_ISL_5852907, EPI_ISL_5852908, EPI_ISL_5852909, EPI_ISL_5852910, EPI_ISL_5852911, EPI_ISL_5852912, EPI_ISL_5852913, EPI_ISL_5852914, EPI_ISL_5852915, EPI_ISL_5852916, EPI_ISL_5852917, EPI_ISL_5852918, EPI_ISL_5852919, EPI_ISL_5852920, EPI_ISL_5852921, EPI_ISL_5852922, EPI_ISL_5852923, EPI_ISL_5852924, EPI_ISL_5852925, EPI_ISL_5852926, EPI_ISL_5859548. (www.gisaid.org).

### Phylogenetic analysis

We downloaded all complete high-quality SARS-CoV-2 genomes from Ukraine from GISAID database on September 30^th^ 2021. All Ukrainian sequences, from GISAID and generated in this study, were aligned using MAFFT. We masked all problematic sites in the alignment as suggested by De Maio et al (https://virological.org/t/masking-strategies-for-sars-cov-2-alignments/480). A maximum likelihood phylogenetic tree was built with IQTREE2 program under the general time reversible model allowing for rate heterogeneity among sites and proportion of invariable sites (GTR+G+I). Pango lineage assignments were determined using the Pangolin nomenclature tool [42]. Mutation calling and sequence quality were analysed using Nextclade (https://clades.nextstrain.org/) and Geneious Prime 2021.2.2 software (Biomatters, New Zealand). Relative distribution of dominant lineages and mutation heatmap was made using R Studio with the ggplot2 package.

### Phylogeographic analysis

To establish the number and the source country of the Delta variant introductions into Ukraine, we have downloaded all complete high-quality with available sampling date Delta variant sequences sampled between January 1^st^ and July 20^th^ available on GISAID on September 30^th^ (N=310,028). We then down-sampled the number of sequences in all location to 12, except for the countries with top-10 number of Delta variant cases in the world (USA, UK, France, Germany, Denmark, India, Canada, Turkey, Sweden, and Switzerland) [43], countries that share border with Ukraine (Russia, Moldova, Romania, Hungary, Poland, Slovakia, Belarus), and other post-Soviet countries, including the three Baltic nations (Estonia, Latvia, Lithuania), Georgia, and Uzbekistan (no Delta sequences were available from the other post-Soviet countries in Central Asia and Transcaucasia); we included 36 sequences from each of these locations in the analysis. We then applied the *mugration* model implemented in TreeTime [44] to estimate the number and originating locations of Delta variant introductions in Ukraine. The *mugration* model assumes countries of origin as discreet states and considers the virus spread between these states as a general time-reversible process.

## Supplementary Material

Supplement 1

Supplement 2

Supplement 3

Supplement 4

Supplement 5

## Figures and Tables

**Table 1:** Samples characterisation and the key amino acid mutation pattern of the sequences.

#	GISAID accession number	Gender	Pango lineage/Variant	Key AA Mutations							
				ORF1a	ORF1b	S	ORF3a	M	ORF7a	ORF8	N
1	EPI_ISL_5852904	M	AY.4 / Delta	-	P314L, P1000L	T19R, E156−, F157−, R158G, L452R, T478K, D614G, P681R, D950N	S26L	I82T	V82A, T120I	-	D63G, R203M, D377Y
2	EPI_ISL_5859548	M	AY.4 / Delta	-	P314L, P1000L	T19R, E156−, F157−, R158G, L452R, T478K, D614G, P681R, D950N	S26L	I82T	-	-	D63G, R203M, D377Y
3	EPI_ISL_5852905	F	AY.4 / Delta	-	P314L, P1000L	T19R, E156−, F157−, R158G, L452R, T478K, D614G, P681R, D950N	S26L	I82T	V82A, T120I	-	D63G, R203M, D377Y
4	EPI_ISL_5852906	F	B.1.1.7 / Alpha	T1001I, A1708D, I2230T, S3675−, G3676−, F3677-	P314L	H69−, V70−, Y144−, N501Y, A570D, D614G, P681H, T716I, S982A, D1118H	-	-	-	Q27*, R52I, Y73C	D3L, R203K, G204R, S235F
5	EPI_ISL_5852907	M	B.1.1.7 +E484K / Alpha	T1001I, A1708D, I2230T, S3675−, G3676−, F3677-	P314L	H69−, V70−, E484K, N501S, A570D, D614G, P681H, T716I, S982A, D1118H	-	-	-	Q27*, R52I, Y73C	D3L
6	EPI_ISL_5852908	F	AY.4 / Delta	-	P314L, P1000L	T19R, E156−, F157−, R158G, L452R, T478K, D614G, P681R, D950N	S26L	I82T	V82A, T120I	-	D63G, R203M, D377Y
7	EPI_ISL_5852909	F	AY.4 / Delta	-	P314L, P1000L	T19R, E156−, F157−, R158G, L452R, T478K, D614G, P681R, D950N	S26L	I82T	V82A, T120I	-	D63G, R203M, D377Y
8	EPI_ISL_5852910	F	AY.4 / Delta	-	P314L, P1000L	T19R, E156−, F157−, R158G, L452R, T478K, D614G, P681R, D950N	S26L	I82T	V82A, T120I	-	D63G, R203M, D377Y
9	EPI_ISL_5852911	M	AY.36 / Delta	-	P314L, P1000L	T19R, E156−, F157−, R158G, L452R, T478K, D614G, P681R, D950N	S26L	I82T	V82A, T120I	-	D63G, R203M, D377Y
10	EPI_ISL_5852912	F	AY.5 / Delta	-	P314L, P1000L	T19R, E156−, F157−, R158G, L452R, T478K, D614G, P681R, D950N	S26L	I82T	V82A, T120I	-	D63G, D377Y
11	EPI_ISL_5852913	F	AY.4 / Delta	-	P314L, P1000L	T19R, E156−, F157−, R158G, L452R, T478K, D614G, P681R, D950N	S26L	I82T	V82A, T120I	-	D63G, R203M, D377Y
12	EPI_ISL_5852914	F	AY.24 / Delta	-	P314L, P1000L	T19R, E156−, F157−, R158G, L452R, T478K, D614G, P681R, D950N	S26L	I82T	V82A	-	D63G, D377Y
13	EPI_ISL_5852915	F	AY.4 / Delta	-	P314L, P1000L	T19R, E156−, F157−, R158G, L452R, T478K, D614G, P681R, D950N	S26L	I82T	V82A, T120I	-	D63G, R203M, D377Y
14	EPI_ISL_5852916	F	AY.4 / Delta	-	P314L, P1000L	T19R, E156−, F157−, R158G, L452R, T478K, D614G, P681R, D950N	S26L	I82T	V82A, T120I	-	D63G, R203M, D377Y
15	EPI_ISL_5852917	F	AY.4 / Delta	-	P314L, P1000L	T19R, E156−, F157−, R158G, L452R, T478K, D614G, P681R, D950N	S26L	I82T	V82A, T120I	-	D63G, R203M, D377Y
16	EPI_ISL_5852918	M	AY.33 / Delta	-	P314L, P1000L	T19R, E156−, F157−, R158G, L452R, T478K, D614G, P681R, D950N	S26L	I82T	V82A, T120I	-	D63G, R203M, D377Y
17	EPI_ISL_5852919	M	AY.36 / Delta	-	P314L, P1000L	T19R, E156−, F157−, R158G, L452R, T478K, D614G, P681R, D950N	S26L	I82T	V82A, T120I	-	D63G, R203M, D377Y
18	EPI_ISL_5852920	F	AY.33 / Delta	-	P314L, P1000L	T19R, E156−, F157−, R158G, L452R, T478K, D614G, P681R, D950N	S26L	I82T	V82A, T120I	-	D63G, R203M, D377Y
19	EPI_ISL_5852921	F	AY.23 / Delta	-	P314L, P1000L	T19R, L452R, T478K, D614G, P681R, D950N	S26L	I82T	V82A, T120I	-	D63G
20	EPI_ISL_5852922	F	AY.4 / Delta	-	P314L, P1000L	T19R, E156−, F157−, R158G, L452R, T478K, D614G, P681R, D950N	S26L	I82T	V82A, T120I	-	D63G, R203M, D377Y
21	EPI_ISL_5852923	F	AY.4 / Delta	-	P314L, P1000L	T19R, E156−, F157−, R158G, L452R, T478K, D614G, P681R, D950N	S26L	I82T	V82A, T120I	-	D63G, R203M, D377Y
22	EPI_ISL_5852924	F	AY.4 / Delta	-	P314L, P1000L	T19R, L452R, T478K, D614G, P681R, D950N	S26L	I82T	V82A, T120I	-	D63G, D377Y
23	EPI_ISL_5852925	M	AY.4 / Delta	-	P314L, P1000L	T19R, E156−, F157−, R158G, L452R, T478K, D614G, P681R, D950N	S26L	I82T	V82A, T120I	-	D63G, R203M, D377Y
24	EPI_ISL_5852926	M	B.1.1.7 / Alpha	T1001I, A1708D, S3675−, G3676−, F3677-	P314L	H69−, V70−, Y144−, N501Y, A570D, D614G, P681H, T716I, S982A, D1118H	-	-	-	Q27*, R52I, Y73C	D3L

## Data Availability

Sequence accession numbers for the consensus genomes in GISAID are as follows: EPI_ISL_5852904, EPI_ISL_5852905, EPI_ISL_5852906, EPI_ISL_5852907, EPI_ISL_5852908, EPI_ISL_5852909, EPI_ISL_5852910, EPI_ISL_5852911, EPI_ISL_5852912, EPI_ISL_5852913, EPI_ISL_5852914, EPI_ISL_5852915, EPI_ISL_5852916, EPI_ISL_5852917, EPI_ISL_5852918, EPI_ISL_5852919, EPI_ISL_5852920, EPI_ISL_5852921, EPI_ISL_5852922, EPI_ISL_5852923, EPI_ISL_5852924, EPI_ISL_5852925, EPI_ISL_5852926, EPI_ISL_5859548.
