# Supplementary material for: Tracking SARS-COV-2 Variants Using Nanopore Sequencing in Ukraine in Summer 2021
Source: Res Sq. 2021 Nov 30:rs.3.rs-1044446. Preprint. [Version 1] doi: 10.21203/rs.3.rs-1044446/v1 (PMC8647652; doi:10.21203/rs.3.rs-1044446/v1)
Supplement: Supplement 1 [file 379a11524df4e50c709db724.docx]

**Supplementary text**

**Sequence quality**

Amplicon dropouts (absent regions regardless of coverage) were observed in most samples, leading to weak or missing regions (<20X read depth coverage), particularly in samples #10, 19, and 22. Notably, amplicon 72 dropout, which covers the beginning of the Spike gene, was detected in all 21 Delta sequences, but not in the 3 Alpha sequences. The second major amplicon 64 (ORF1ab) dropout was detected for 12 Delta sequences. Individual random amplicon dropouts were detected in a few samples with coverage <97% in the ORF1ab, S, ORF6, ORF8 and N genes.

**Most common aa mutations**

All Alpha variant sequences (samples #4, #5 and #24) possess the same aa mutations in ORF1a: T1001I, A1708D, S3675-, G3676-, F3677-; in S: H69-, V70-, Y144-, N501Y, A570D, P681H, T716I, S982A, D1118H; in ORF8: Q27*, R52I, Y73C; and in N: D3L. The mutation I2230T in ORF1a was found only in two sequences (samples #4 and #5). Only sample #4 has gained the common Alpha mutations such as R203K, G204R, S235F in N protein. For all 21 Delta variant sequences the following mutations were identified: P1000L in ORF1b; T19R, L452R, T478K, P681R, D950N in S protein; S26L in ORF3a; I82T in M; D63G in N. The other common Delta mutations detected in S protein, such as E156-, F157-, and R158G, were present only in 19 out of 21 sequences and were absent in samples #19 and #22. The R203M and D377Y mutations in N were gained in 17 and 20 sequences, respectively. In ORF7a, the V82A mutation was present in all but one sample (#2) and T120I in all but two samples #2 and #12). The most common co-occurred mutations P314L in ORF1b/RdRp and D614G in S protein were present in all 24 sequences.

**Pango lineages**

From the Oxford Nanopore sequences generated in this study, among the 21 Delta (B.1.617.2-like) variant sequences 6 different Delta sub-lineages were assigned using the PANGO Lineages tool: AY.4 (N=14) and AY.5 (N=1) UK sub-lineages, AY.23 (N=1) predominantly Singapore and Indonesia sub-lineage, AY.24 (N=1) predominantly Indonesia, AY.33 (N=2) sub-lineage circulating mostly in Northwestern Europe, AY.36 (N=2) which is predominantly in Nigeria, UK, and USA. Three out of 24 sequences belong to the Alpha (B.1.1.7-like) variant, and one of them was assigned as B.1.1.7-like+ E484K sub-variant.
