# Supplementary material for: Tracking SARS-COV-2 Variants Using Nanopore Sequencing in Ukraine in Summer 2021
Source: Res Sq. 2021 Nov 30:rs.3.rs-1044446. Preprint. [Version 1] doi: 10.21203/rs.3.rs-1044446/v1 (PMC8647652; doi:10.21203/rs.3.rs-1044446/v1)
Supplement: Supplement 3 [file ebd8373b9a328584ea7ef9e0.pdf]

Table S4. Acknowledgement GISAID table.

We gratefully acknowledge the following Authors from the Originating laboratories responsible for obtaining the specimens, as well as the Submitting laboratories where the genome data were generated and shared via GISAID, on which this research is based.
All Submitters of data may be contacted directly via www.gisaid.org

Accession ID	Originating Laboratory	Submitting Laboratory	Authors
EPI_ISL_1298483, EPI_ISL_1298488	Bogorodchany CRH	The Institute of Molecular Biology and Genetics of NASU	M.Tukalo et al.
EPI_ISL_1298474	Burshtyn CCH	The Institute of Molecular Biology and Genetics of NASU	M.Tukalo et al.
EPI_ISL_576146, EPI_ISL_576147, EPI_ISL_576148, EPI_ISL_576149	Department of Respiratory & Other Viral Infections of L.V. Gromashevsky Institute of Epidemiology & Infectious Diseases NAMS of Ukraine	Department of Respiratory & Other Viral Infections of L.V. Gromashevsky Institute of Epidemiology & Infectious Diseases NAMS of Ukraine, JSC "Farmak"	Alla Mironenko; Andriy Goy; Ihor Kravchuk; Larysa Radchenko; Liudmyla Bolotova; Nataliia Teteriuk
EPI_ISL_4260382, EPI_ISL_4260384, EPI_ISL_4260386, EPI_ISL_4260388, EPI_ISL_4260390, EPI_ISL_4260392, EPI_ISL_4260393, EPI_ISL_4260395, EPI_ISL_4260396, EPI_ISL_4260398, EPI_ISL_4260400, EPI_ISL_4260402, EPI_ISL_4260404, EPI_ISL_4260406, EPI_ISL_4260408, EPI_ISL_4260410, EPI_ISL_4260411, EPI_ISL_4260413, EPI_ISL_4260414, EPI_ISL_4260416, EPI_ISL_4260418, EPI_ISL_4260420, EPI_ISL_4260424, EPI_ISL_4260427, EPI_ISL_4260429, EPI_ISL_4260430, EPI_ISL_4260432, EPI_ISL_4260433, EPI_ISL_4260435, EPI_ISL_4260436, EPI_ISL_4260438, EPI_ISL_4260440, EPI_ISL_4260442, EPI_ISL_4260444, EPI_ISL_4260445, EPI_ISL_4260449, EPI_ISL_4260450			
see above	Department of Respiratory and other Viral Infections of L.V. Gromashevsky Institute of Epidemiology and Infectious Diseases NAMS of Ukarine	CNR Virus des Infections Respiratoires - France SUD	Alla Mironenko; Antonin Bal; Bruno Lina; Gregory Destras; Gwendolyne Burfin; Hadrien Regue; Larysa Radchenko; Laurence Josset; Martine Valette; Nataliia Teteriuk; Quentin Semanas
EPI_ISL_582509	Department of Respiratory and other Viral Infections of L.V.Gromashevsky Institute of Epidemiology & Infectious Diseases NAMS of Ukraine	Department of Respiratory and other Viral Infections of L.V.Gromashevsky Institute of Epidemiology & Infectious Diseases NAMS of Ukraine, JSC "Farmak"	Alla Mironenko; Andriy Goy; Ihor Kravchuk; Larysa Radchenko; Ludmyla Bolotova; Nataliia Teteriuk
EPI_ISL_582510	Department of Respiratory and other Viral Infections of L.V.Gromashevsky Institute of Epidemiology & Infectious Diseases NAMS of Ukraine	Department of Respiratory and other Viral Infections of L.V.Gromashevsky Institute of Epidemiology & Infectious Diseases NAMS of Ukraine, JSC "Farmak"	Alla Mironenko; Andriy Goy; Ihor Kravchuk; Larysa Radchenko; Ludmyla Bolotova; Nataliia Teteriuk
EPI_ISL_582511, EPI_ISL_582512, EPI_ISL_582513, EPI_ISL_654819, EPI_ISL_654820, EPI_ISL_979970, EPI_ISL_979971, EPI_ISL_979972, EPI_ISL_979973, EPI_ISL_1121990, EPI_ISL_1121991, EPI_ISL_1121992, EPI_ISL_1122014, EPI_ISL_1315427, EPI_ISL_1315475, EPI_ISL_1315476, EPI_ISL_1315477, EPI_ISL_1315478			
see above	Department of Respiratory and other Viral Infections of L.V.Gromashevsky Institute of Epidemiology & Infectious Diseases NAMS of Ukraine	Department of Respiratory and other Viral Infections of L.V.Gromashevsky Institute of Epidemiology & Infectious Diseases NAMS of Ukraine, JSC "Farmak"	Alla Mironenko; Andriy Goy; Ihor Kravchuk; Larysa Radchenko; Ludmyla Bolotova; Nataliia Teteriuk
EPI_ISL_654818	Department of Respiratory and other Viral Infections of L.V.Gromashevsky Institute of Epidemiology & Infectious Diseases NAMS of Ukraine	Department of Respiratory and other Viral Infections of L.V.Gromashevsky Institute of Epidemiology & Infectious Diseases NAMS of Ukraine, JSC "Farmak"	Alla Mironenko; Andriy Goy; Ihor Kravchuk; Larysa Radchenko; Ludmyla Bolotova; Nataliia Teteriuk
EPI_ISL_953938, EPI_ISL_954750	Diagen	Diagen	Koliada O
EPI_ISL_3253254, EPI_ISL_3253255, EPI_ISL_3253256, EPI_ISL_3253257, EPI_ISL_3253258, EPI_ISL_3253259, EPI_ISL_3253260, EPI_ISL_3253261, EPI_ISL_3253262, EPI_ISL_3253263, EPI_ISL_3253264, EPI_ISL_3253265, EPI_ISL_3253266, EPI_ISL_3253267, EPI_ISL_3253268, EPI_ISL_3253269, EPI_ISL_3253270, EPI_ISL_3253271, EPI_ISL_3253272, EPI_ISL_3253273, EPI_ISL_3253274, EPI_ISL_3253275, EPI_ISL_3253276, EPI_ISL_3253277, EPI_ISL_3253278, EPI_ISL_3253279, EPI_ISL_3253280, EPI_ISL_3253281, EPI_ISL_3253282, EPI_ISL_3253283, EPI_ISL_3253284, EPI_ISL_3253285, EPI_ISL_3253286, EPI_ISL_3253287, EPI_ISL_3253288, EPI_ISL_3253289, EPI_ISL_3253290, EPI_ISL_3253291, EPI_ISL_3253292, EPI_ISL_3253293, EPI_ISL_3253294, EPI_ISL_3253295, EPI_ISL_3253296, EPI_ISL_3253297, EPI_ISL_3314947, EPI_ISL_3314948, EPI_ISL_3314949			
see above	Dutch COVID-19 response team	National Institute for Public Health and the Environment (RIVM)	Adam Meijer; AnneMarie van den Brandt; Annelies Kroneman; Bas van der Veer; Chantal Reusken; Dennis Schmitz; Dirk Eggink; Euníce Then; Florian Zwagemaker; Harry Vennema; Jeroen Cremer; Karim Hajji; Kim Freriks; Lisa Wijsman; Lynn Aarts; Melissa van Tuil; Ryanne Jaarsma; Sanne Bos; Sharon van den Brink; on behalf of the national COVID-19 response team
EPI_ISL_1298476, EPI_ISL_1298477, EPI_ISL_1298481	Ivano-Frankivsk CCH#1	The Institute of Molecular Biology and Genetics of NASU	M.Tukalo et al.
EPI_ISL_1298487	Kalush CRH	The Institute of Molecular Biology and Genetics of NASU	M.Tukalo et al.
EPI_ISL_1298475, EPI_ISL_1298478, EPI_ISL_1298479, EPI_ISL_1298485, EPI_ISL_1298486	Kosov CRH	The Institute of Molecular Biology and Genetics of NASU	M.Tukalo et al.
EPI_ISL_1298480, EPI_ISL_1298484	Nadvirna CRH	The Institute of Molecular Biology and Genetics of NASU	M.Tukalo et al.
EPI_ISL_512597, EPI_ISL_512598, EPI_ISL_512599, EPI_ISL_512600, EPI_ISL_512601, EPI_ISL_512602, EPI_ISL_512603, EPI_ISL_512604, EPI_ISL_512605, EPI_ISL_512606, EPI_ISL_512607, EPI_ISL_512608, EPI_ISL_512609, EPI_ISL_512610, EPI_ISL_512611, EPI_ISL_512612, EPI_ISL_512613, EPI_ISL_512614, EPI_ISL_512615, EPI_ISL_512616, EPI_ISL_512617, EPI_ISL_512618, EPI_ISL_512619, EPI_ISL_512620, EPI_ISL_512621, EPI_ISL_512622, EPI_ISL_512623, EPI_ISL_512624, EPI_ISL_512625, EPI_ISL_512626, EPI_ISL_512627, EPI_ISL_512628, EPI_ISL_512629, EPI_ISL_512630, EPI_ISL_512631, EPI_ISL_512632, EPI_ISL_512633, EPI_ISL_512634, EPI_ISL_512635, EPI_ISL_512636, EPI_ISL_512637, EPI_ISL_512638, EPI_ISL_512639, EPI_ISL_512640, EPI_ISL_512641, EPI_ISL_512642, EPI_ISL_512643, EPI_ISL_512644, EPI_ISL_512810			
see above	National Laboratory for Influenza/Virology reference laboratory, Public Health Center of the Ministry of Health of Ukraine	Respiratory Virus Unit, Microbiology Services Colindale, Public Health England	Dr. Iryna Demchyshyna; PHE Covid Sequencing Team
EPI_ISL_4440036, EPI_ISL_4440039, EPI_ISL_4440042, EPI_ISL_4440044, EPI_ISL_4440046, EPI_ISL_4440048, EPI_ISL_4440051, EPI_ISL_4440053, EPI_ISL_4440055, EPI_ISL_4440057, EPI_ISL_4440060, EPI_ISL_4440062, EPI_ISL_4440064, EPI_ISL_4440066, EPI_ISL_4440068, EPI_ISL_4440071, EPI_ISL_4440073, EPI_ISL_4440075, EPI_ISL_4440077, EPI_ISL_4440078, EPI_ISL_4440080, EPI_ISL_4440082, EPI_ISL_4440084, EPI_ISL_4440087, EPI_ISL_4440089, EPI_ISL_4440091, EPI_ISL_4440092, EPI_ISL_4440093, EPI_ISL_4440095, EPI_ISL_4440097, EPI_ISL_4440099, EPI_ISL_4440101, EPI_ISL_4440103			
see above	Public Health Center of MHU	The Institute of Molecular Biology and Genetics of NASU	M.Tukalo et al.
EPI_ISL_1112293, EPI_ISL_1112294, EPI_ISL_1112295, EPI_ISL_1112296, EPI_ISL_1112297, EPI_ISL_1112298, EPI_ISL_1112299, EPI_ISL_1112300, EPI_ISL_1112301, EPI_ISL_1112302, EPI_ISL_1112303, EPI_ISL_1112304, EPI_ISL_1112305, EPI_ISL_1112306, EPI_ISL_1112307, EPI_ISL_1112308, EPI_ISL_1112309, EPI_ISL_1112310, EPI_ISL_1112311, EPI_ISL_1112312, EPI_ISL_1112313, EPI_ISL_1112314, EPI_ISL_1112315, EPI_ISL_1112316, EPI_ISL_1112317, EPI_ISL_1112318, EPI_ISL_1112319, EPI_ISL_1112320, EPI_ISL_1112321, EPI_ISL_1112322, EPI_ISL_1112323, EPI_ISL_1112324, EPI_ISL_1112325, EPI_ISL_1112326, EPI_ISL_1112327, EPI_ISL_1112328, EPI_ISL_1112329, EPI_ISL_1112330, EPI_ISL_1112331, EPI_ISL_1112332, EPI_ISL_1112333, EPI_ISL_1112334, EPI_ISL_1112335, EPI_ISL_1112336, EPI_ISL_1112337, EPI_ISL_1112338, EPI_ISL_1112339, EPI_ISL_1112753			
see above	Public Health Center of Ukraine	Charité Universitätsmedizin Berlin, Institute of Virology	Barbara Mühlemann; Christian Drosten; Ihor Kuzin; Iryna Demchyshyna; Julia Schneider; Jörn Beheim-Schwarzbach; Liudmyla Chernenko; Roman Rodyna; Talitha Veith; Terry Jones; Victor M Corman
EPI_ISL_2934571, EPI_ISL_2934572, EPI_ISL_2934575, EPI_ISL_2966511, EPI_ISL_2966513	SE Kyiv CDC MHU	The Institute of Molecular Biology and Genetics of NASU	M.Tukalo et al.
EPI_ISL_2934569, EPI_ISL_2934570, EPI_ISL_2934573, EPI_ISL_2934574, EPI_ISL_2966512	SECP MHU	The Institute of Molecular Biology and Genetics of NASU	M.Tukalo et al.
EPI_ISL_3262118, EPI_ISL_3262119, EPI_ISL_3262120, EPI_ISL_3262121, EPI_ISL_3262122, EPI_ISL_3262123, EPI_ISL_3262124, EPI_ISL_3262125, EPI_ISL_3262126, EPI_ISL_3262127, EPI_ISL_3262128, EPI_ISL_3262129, EPI_ISL_3262130, EPI_ISL_3262131, EPI_ISL_3262132, EPI_ISL_3262133, EPI_ISL_3262134, EPI_ISL_3262135, EPI_ISL_3262136, EPI_ISL_3262137, EPI_ISL_3262138			
see above	SI «Public Health Center of MHU»	The Institute of Molecular Biology and Genetics of NASU	M.Tukalo et al.
EPI_ISL_1495111, EPI_ISL_1495112, EPI_ISL_1495113, EPI_ISL_1495114, EPI_ISL_1495115, EPI_ISL_1495116, EPI_ISL_1495117, EPI_ISL_1495118, EPI_ISL_1495119, EPI_ISL_1495120, EPI_ISL_1495121, EPI_ISL_1495122, EPI_ISL_1495123, EPI_ISL_1495124, EPI_ISL_1495125, EPI_ISL_1495126, EPI_ISL_1495127, EPI_ISL_1495128, EPI_ISL_1495129, EPI_ISL_1495130, EPI_ISL_1495131, EPI_ISL_1495132, EPI_ISL_1495133, EPI_ISL_1495134, EPI_ISL_1495135, EPI_ISL_1495136, EPI_ISL_1495137, EPI_ISL_1495138, EPI_ISL_1495139, EPI_ISL_1495140, EPI_ISL_1495141, EPI_ISL_1495142, EPI_ISL_1495143			
see above	State Institution «Public Health Center of Ministry of Health of Ukraine»	Robert Koch Institute, ZBS1 Highly Pathogenic Viruses, Berlin, Germany	Andreas Nitsche; Annika Brinkmann; Iryna Demchyshyna; Janine Michel; Liudmyla Chernenko; Roman Rodyna; Steven Uddin
EPI_ISL_1298482	Tysmenytsia CH	The Institute of Molecular Biology and Genetics of NASU	M.Tukalo et al.

Accession ID	Originating Laboratory	Submitting Laboratory	Authors
EPI_ISL_733077, EPI_ISL_733078, EPI_ISL_733151, EPI_ISL_733433, EPI_ISL_733452, EPI_ISL_1400527, EPI_ISL_1400557			
see above	HELIX LLC	WHO National Influenza Centre Russian Federation	Andrey Komissarov; Anna Ivanova; Artem Fadeev; Daria Danilenko; Dmitry Bazhenov; Dmitry Lioznov; Elena Nabieva; Georgii Bazykin; Ksenia Safina; Kseniya Komissarova
EPI_ISL_3932047, EPI_ISL_3996685, EPI_ISL_3996686, EPI_ISL_3996687, EPI_ISL_3996688	Molecular Diagnostic Laboratory of Federal Budget Institution of Science at the Central Research Institute of Epidemiology of the Federal Service on Customers' Rights Protection and Human Well-being Surveillance	Group of Genomics and Postgenomic Technologies of Central Research Institute of Epidemiology	Akimkin V.G.; Berlina Y.Y.; Buharina A.Y.; Kaptelova V.V.; Kondrasheva L.Y.; Korneenko E.V.; Nadtoka M.I.; Roev G.V.; Saenko S.S.; Samoilov A.E.; Shipulina O.Y.; Sinitsyn S.O.; Smirnova Y.S.; Speranskaya A.S.; Vyhodceva A.V.
EPI_ISL_2031957	Molecular diagnostic laboratory of Federal Budget Institution of Science "Central Research Institute of Epidemiology" of The Federal Service on Customers' Rights Protection and Human Well-being Surveillance	Group of Genomics and Postgenomic Technologies of Central Research Institute of Epidemilology	Akimkin V.G.; Kaptelova V.V.; Kondrasheva L.Y.; Korneenko E.V.; Nadtoka M.I.; Saenko S.S.; Samojlov A.E.; Shipulina O.Y.; Sinicyn S.O.; Smirnova Y.S.; Speranskaya A.S.; Tivanova E.V.
EPI_ISL_3101330, EPI_ISL_3101347	Molecular diagnostic laboratory of Federal Budget Institution of Science "Central Research Institute of Epidemiology" of The Federal Service on Customers' Rights Protection and Human Well-being Surveillance	Group of Genomics and Postgenomic Technologies of Central Research Institute of Epidemiology	Akimkin V.G.; Berlina Y.Y.; Bulanenko V.P.; Cherkashina A.S.; Dohoyan A.Y.; Golubeva A.G.; Kaptelova V.V.; Kondrasheva L.Y.; Korneenko E.V.; Nadtoka M.I.; Saenko S.S.; Samoilov A.E.; Shipulina O.Y.; Smirnova Y.S.; Solovieva E.D.; Speranskaya A.S.; Valdohina A.V.; Zotova M.I.; Zuev S.N.
EPI_ISL_3122988, EPI_ISL_3122989, EPI_ISL_3122990, EPI_ISL_3122991, EPI_ISL_3122992, EPI_ISL_3122993, EPI_ISL_3122994, EPI_ISL_3122995, EPI_ISL_3122996, EPI_ISL_3122997, EPI_ISL_3122998, EPI_ISL_3122999, EPI_ISL_3123000, EPI_ISL_3123001, EPI_ISL_3123002, EPI_ISL_3123003, EPI_ISL_3123004, EPI_ISL_3123005, EPI_ISL_3123006, EPI_ISL_3123007, EPI_ISL_3123008			
see above	Republican Children’s Clinical Infectious Diseases Hospital	WHO National Influenza Centre Russian Federation	Alexey Masharsky; Andrey Komissarov; Artem Fadeev; Daria Danilenko; Dmitry Lioznov; Elena Nabieva; Georgii Bazykin; Kirill Varchenko; Ksenia Safina; Kseniya Komissarova; Maria Baturova; Maria Pisareva; Mikhail Bakaev; Nikita Yolshin; Oula Mansour; Tamila Musaeva; Veronika Eder
EPI_ISL_428901	State Research Center of Virology and Biotechnology VECTOR, Department of Collection of Microorganisms	State Research Center of Virology and Biotechnology VECTOR, Department of Collection of Microorganisms	Alexander N. Shvalov; Elena V. Gavrilova; Oleg V. Pyankov; Rinat A. Maksyutov; Sergey A. Bodnev; Tatyana V. Tregubcha

Accession ID	Originating Laboratory	Submitting Laboratory	Authors
EPI_ISL_4536678, EPI_ISL_4536683, EPI_ISL_4536741, EPI_ISL_4536770, EPI_ISL_4558178	Molecular diagnostic laboratory of Federal Budget Institution of Science "Central Research Institute of Epidemiology" of The Federal Service on Customers' Rights Protection and Human Well-being Surveillance	Group of Genomics and Postgenomic Technologies of Central Research Institute of Epidemiology	Akimkin V.G.; Buharina A.Y.; Kondrasheva L.Y.; Korneenko E.V.; Nadtoka M.I.; Roev G.V.; Samoilov A.E.; Shipulina O.Y.; Sinitsyn S.O.; Smirnova Y.S.; Speranskaya A.S.; Svetlichnyj D.V.; Vyhodceva A.V.
