# Supplementary figures and images for: Tracking SARS-COV-2 Variants Using Nanopore Sequencing in Ukraine in Summer 2021
Source: Res Sq. 2021 Nov 30:rs.3.rs-1044446. Preprint. [Version 1] doi: 10.21203/rs.3.rs-1044446/v1 (PMC8647652; doi:10.21203/rs.3.rs-1044446/v1)

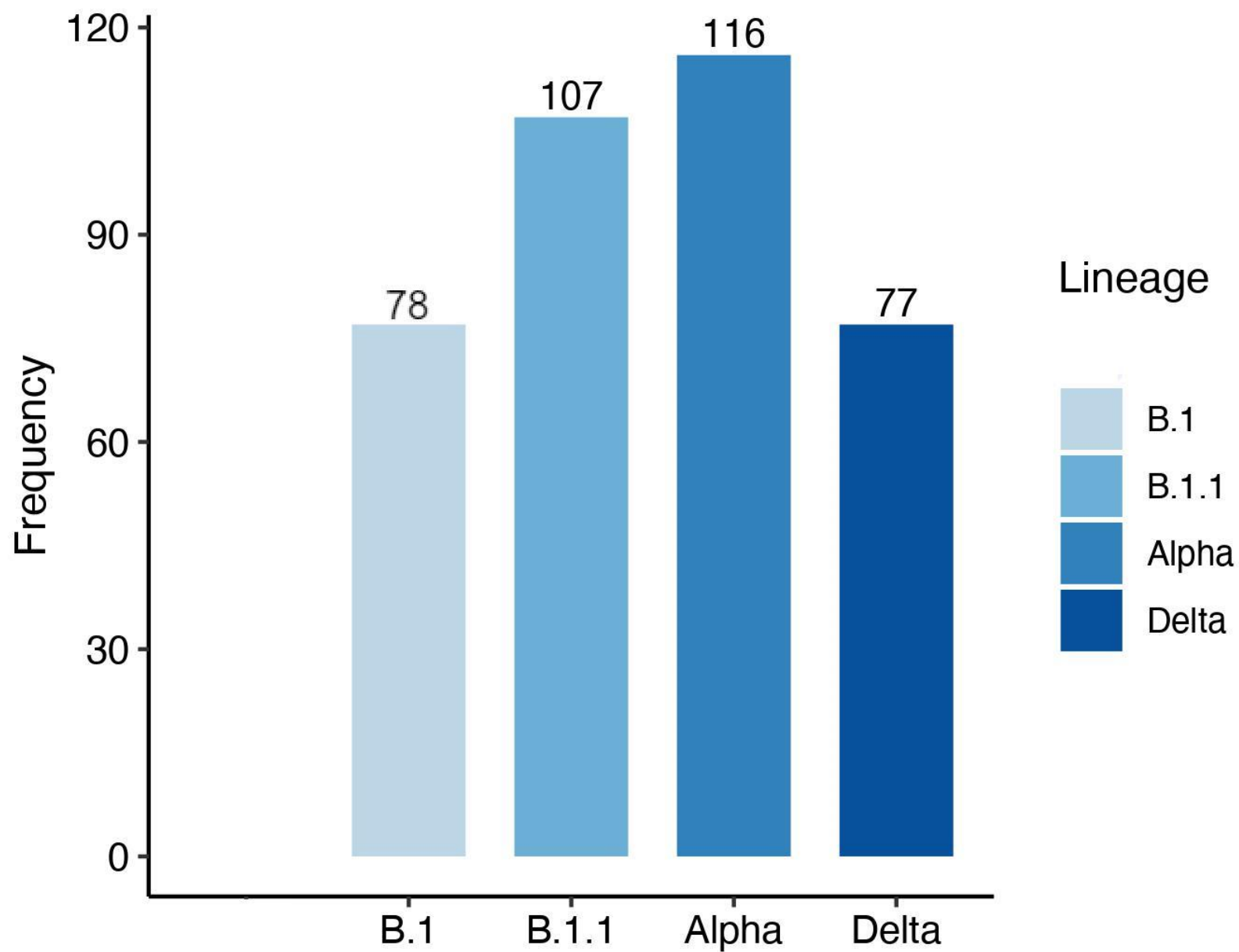

**Figure S1.** The distribution of SARS-CoV-2 predominant lineages and variants of concern in Ukraine.

Supplement: Supplement 4 [file b007fea652a68da9ae46ba22.pdf]
