# Supplementary material for: Tracking SARS-COV-2 Variants Using Nanopore Sequencing in Ukraine in Summer 2021
Source: Res Sq. 2021 Nov 30:rs.3.rs-1044446. Preprint. [Version 1] doi: 10.21203/rs.3.rs-1044446/v1 (PMC8647652; doi:10.21203/rs.3.rs-1044446/v1)

Figure S2. Maximum likelihood phylogenetic tree of the SARS-CoV-2 Delta variant sequences sampled in June-July in Ukraine. Red circles indicate the SARS-C0V-2 Delta genomes from Ukraine. The colors of the arrows indicate countries from which the Delta lineages were introduced.

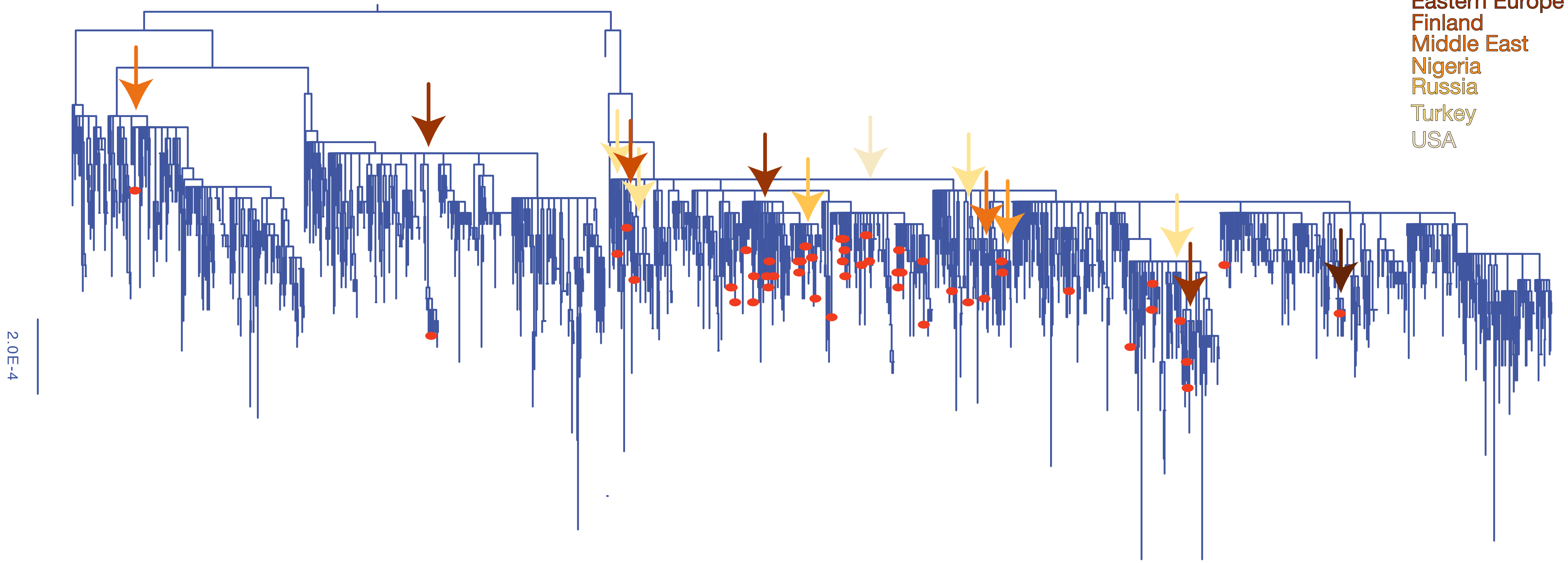

Supplement: Supplement 5 [file 64eca6e16e60a95eeee12476.pdf]
